# Peripheral Blood Mononuclear Cell Transcriptome of Dairy Cows Naturally Infected with Bovine Leukemia Virus

**DOI:** 10.3390/pathogens13100885

**Published:** 2024-10-11

**Authors:** Tanner F. Scull, Clarissa Strieder-Barboza, Oscar J. Benitez

**Affiliations:** 1 Department of Veterinary Sciences, Davis College of Agricultural Sciences and Natural Resources, Texas Tech University, Lubbock, TX 79409, USA; tanner.scull@ttu.edu (T.F.S.); cstriede@ttu.edu (C.S.-B.); 2 School of Veterinary Medicine, Texas Tech University, Amarillo, TX 79106, USA

**Keywords:** BLV, transcriptome, PBMC, cattle, lymphocyte, gene expression, immune dysregulation

## Abstract

The current literature has identified many abnormalities in the immune expression of cows infected with the bovine leukemia virus (BLV). These studies have focused on individual cell, gene, or protein expression, failing to provide a comprehensive understanding of the changes in immune expression in animals with BLV. To identify the overall alterations in immune expression during BLV infection, the transcriptomes of the peripheral blood mononuclear cells (PBMCs) of cows seropositive or seronegative for BLV antibodies were sequenced. Whole blood samples were collected from 20 dairy cows and screened for BLV antibodies and PCR was used to quantify the proviral load of the samples. PBMCs were separated from whole blood using density gradient centrifugation from which RNA was isolated and sequenced. Three seropositive samples (BLV+; *n* = 3), including one of each PVL category, low (*n* = 1), moderate (*n* = 1), and high (*n* = 1), and three seronegative samples (BLV−; *n* = 3) were sequenced for differential gene expression analysis. The results showed major differences in the transcriptome profiles of the BLV+ and BLV− PBMCs and revealed a wide variety of immunological pathways affected by BLV infection. Our results suggest that disease state and PBMC gene expression vary depending on BLV proviral load levels and that BLV causes the suppression of normal immune responses and influences B and T cell gene expression, resulting in immune dysfunction.

## 1. Introduction

Bovine leukemia virus (BLV) is an oncogenic B-lymphocytotropic delta-retrovirus closely related to human T cell leukemia viruses [1]. Preferentially infecting B cells, BLV integrates into the DNA of the infected cell as a DNA provirus [2], and can lead to the development of enzootic bovine leukosis [3]. The clinical form of the infection, B cell leukemia/lymphoma, develops in only 0.5–10% of the cases. Most infected cattle remain subclinical with 30% developing persistent lymphocytosis (PL), characterized by an increased number of B lymphocytes due to the polyclonal expansion of the affected cells. The remaining 65% of the infections are serologically positive for BLV, but negative for PL, and described as aleukemic [4]. 

The bovine leukemia virus is widespread, affecting over 83% of the dairy herds throughout the United States, and is readily transmitted through contact with blood and other bodily fluids containing provirus-infected lymphocytes. This pathogen is of great economic concern to the dairy industry and is responsible for decreased milk production, shortened lifespan, the occurrence of secondary infections, and other health and management-related costs [5]. While there have been no specific mechanisms identified by which BLV interferes with milk production or longevity, the current literature suggests that immune dysregulation in infected cattle is the root cause. 

The bovine leukemia virus causes systemic immune dysfunction characterized by reduced and irregular cellular immune responses and activities [6,7,8]. This immune dysregulation manifests as alterations in immune cells’ surface protein expression, cytokine expression, and changes in the overall lymphocyte counts and apoptosis rates in infected animals [9,10]. Many instances of immune modulation due to BLV infection have been observed, specifically in B and T cells. For example, infected cows exhibit a reduced humoral response to vaccination, while B cells from cows with PL exhibit a lower expression of lambda immunoglobulin chains. T cells from infected animals display an altered phenotype characterized by a higher expression of negative regulatory proteins LAG3 and TIM3 indicating the suppression of T cell activity. Changes in B cell protein expression, such as increased levels of T cell repressor PD-L1, further point to the suppression of T cell function [11]. 

Currently, proviral load (PVL) quantification, the number of copies of BLV in genomic DNA, is used to categorize the infection state of animals during scientific study [12]. This categorization is utilized because PVL typically correlates with disease progression and the likelihood of transmission [13]. Further, it has been found that cows with high BLV PVL are more susceptible to secondary infections, such as mastitis, and more often display abnormal numbers of lymphocytes compared to cows with low and moderate PVL.

Despite several observed differences between the immune cell expression of BLV-positive and BLV-negative cows, current studies have only investigated specific proteins and inflammatory mediators and have failed to address the systemic changes in immune function and gene expression in lymphocytes and other immune cells and the relationship between PVL and these alterations. The objective of the current study is to identify differences in the transcriptome of the peripheral blood mononuclear cells (PBMCs) of BLV-positive and BLV-negative cows and assess differences based on PVL to provide a more thorough understanding of immune modulation in BLV infection.

## 2. Materials and Methods

### 2.1. Samples

Whole blood samples were collected from twenty mid-lactation Holstein dairy cows (3rd or 4th lactations) at one dairy farm in West Texas. All the animals selected for sampling were under the routine supervision of a veterinarian and had no obvious health issues. The samples were collected via coccygeal venipuncture using Vacutainer needles and EDTA tubes (Becton Dickinson and Company, Franklin Lakes, NJ, USA). After collection, the samples were stored at 4 °C in a temperature-controlled cooler and transported to the laboratory.

### 2.2. Peripheral Blood Mononuclear Cell Isolation

Peripheral blood mononuclear cell isolation was performed using an adapted protocol based on previous methods. The tubes were centrifuged at 900 RCF for 10 min after plasma separation, and the buffy coat was transferred to a 15 mL conical tube and mixed 1:1 in phosphate-buffered saline (PBS) (ThermoScientific, Waltham, MA, USA). The PBS/buffy coat mixture was carefully layered on top of 5 mL of Ficoll-Paque Plus density gradient media (Cytiva, Marlborough, MA, USA) and centrifuged at 900 RCF for 22 min at room temperature. The separated PBMC layer was transferred to a new 15 mL tube containing 10 mL of PBS and centrifuged for 5 min at 250 RCF for washing. The supernatant was removed, the pellet was resuspended in 10 mL of PBS, and again centrifuged for 5 min at 250 RCF for a second washing. The cells were resuspended in 5 mL of warmed Dulbecco’s Modified Eagle Medium containing no phenol red, glucose, glutamine, or sodium pyruvate (ThermoScientific, Waltham, MA, USA).

### 2.3. RNA Purification

Cells were counted using a Corning Cell Counter followed by PBMC RNA purification using the RNeasy^®^ Mini Kit (Qiagen, Germantown, MD, USA). Approximately 5 × 10^6^ cells from each sample were resuspended in 350 µL of buffer RLT for disruption per protocol. RNA extraction was performed according to the RNeasy^®^ Mini Kit Quick-Start Protocol (November 2021). The isolated RNA samples dissolved in RNase-free water were then screened for concentration and purity using a NanoDrop One^c^ Microvolume UV-Vis Spectrophotometer (ThermoScientific). The total RNA dissolved in RNase-free water was stored at −80 °C.

### 2.4. Bovine Leukemia Virus Antibody and Proviral Load Determination

One tube of whole blood from each animal was used by a commercial provider (CentralStar Cooperative, Inc., Lansing, MI, USA) to determine sample seropositivity for BLV (ELISA) and to quantify the PVL of ELISA-positive samples (AntelBio^TM^ STRATA-G^TM^ BLV PCR test). The results showed that 17/20 cows sampled were seropositive for BLV antibodies. The seropositive samples underwent PCR testing to quantify PVL as described by previous work [14], in which PVL was expressed as copies per cell (CPC) and is equivalent to the ratio between proviral BLV copies and Bos Taurus Beta-actin gene copies. PVL was categorized as undetected (0.0 CPC), low (<0.5 CPC), moderate (≤0.5, <1.0 CPC), and high (≥1.0 CPC). The results reported 7 samples as low PVL, 1 as moderate PVL, 1 as high PVL, and 8 as undetected.

### 2.5. Sample Selection, Library Preparation, and Sequencing

RNA samples were selected for sequencing based on the BLV antibody and PVL screening results and included 3 seropositive samples (BLV+; *n* = 3), including 1 of each PVL category, low (*n* = 1), moderate (*n* = 1), high (*n* = 1), and 3 seronegative samples (BLV−; *n* = 3). The RNA samples were sequenced by a commercial provider that prepared a cDNA library from the whole RNA provided. Messenger RNA was purified from the total RNA sample using poly-T oligo-attached magnetic beads. The mRNA was fragmented, and cDNA was synthesized using random hexamer primers and dTTPs followed by adapter ligation, size selection, amplification, and purification. Libraries were sequenced on the Illumina NovaSeq 6000 platform (Illumina, San Deigo, CA, USA) and generated approximately 20 million 150 bp read pairs.

### 2.6. Quality Control and Genome Mapping

The raw reads were stored in the FASTQ format for quality control. Adapter sequences and low-quality reads were filtered from the raw data. The reads were mapped to the Ensembl Bos taurus ARS-UCD1.2 reference genome using the HISAT2 (v2.0.5) alignment program and FeatureCounts (v1.5.0-p3) provided read counts, allowing for gene expression quantification reported in Fragments Per Kilobase of transcript sequence per Millions base pairs sequenced (FPKM). A correlation analysis was performed to assess similarities in the gene expression of the biological replicates within the BLV− group and the BLV+ group. All the within-group squares of the Pearson correlation coefficient were greater than the ENCODE project’s recommended 0.92 for ideal experiment conditions.

### 2.7. Differential Gene Expression Analysis

Differential gene expression analysis was performed using the DESeq2R package (1.20.0). *p*-values were adjusted using Benjamini and Hochberg’s approach for controlling the false discovery rate. The genes with an adjusted *p*-value ≤ 0.05 and Log2 fold-change ≥1 or ≤−1 were denoted as differentially expressed. The differentially expressed genes (DEGs) were pooled for cluster analysis as the differential gene set and mainstream hierarchical clustering was used to cluster the genes based on the FPKM values. The gene expression values were homogenized across rows to obtain relative Z-scores between the samples for each DEG. Functional analysis is a method used to obtain biological insights from the identification of DEGs by analyzing how the genes and their pathways interact to result in a given function. Functional analysis was performed using DEGs via the clusterProfiler R package v3.8.1 [15] for Gene Ontology (GO) enrichment analysis and KEGG pathway enrichment analysis, and all the values were corrected in the clusterProfiler R package to account for gene length bias.

## 3. Results

### 3.1. Differential Gene Expression and Clustering Analysis

**Figure 1:**
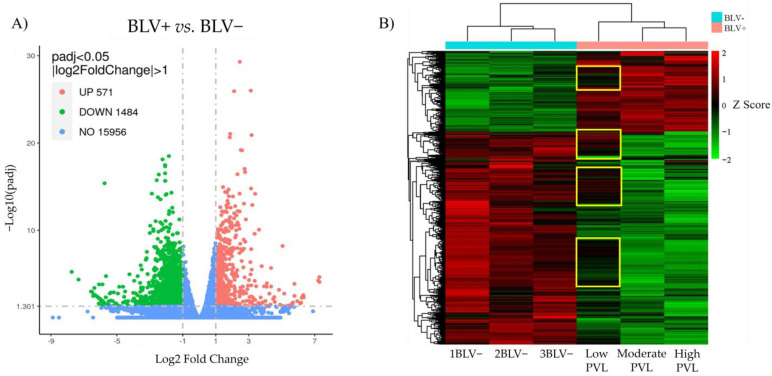
Differential gene expression analysis results. (**A**) Volcano plot displaying all the genes identified after sequencing in terms of −Log10(padj) and Log2 Fold change with thresholds for the DEGs set at >1.301 and <−1 or >1, respectively. The results display 571 upregulated and 1484 downregulated DEG in BLV+ vs. BLV− PBMCs. (**B**) Hierarchical clustering heat map from the results of the FPKM cluster analysis clustered using the log2(FPKM+1) value to compare gene expression between the six samples. Red indicates relative upregulation and green indicates relative downregulation. The yellow boxes highlight the gene clusters in the low-PVL sample that are dissimilar to the other BLV+ samples.

The results showed definitive differences in the transcriptomes of the BLV+ and BLV− PBMCs. A total of 18,011 genes were identified; of these, 571 were upregulated and 1484 were downregulated in BLV+ vs. BLV− PBMCs (Figure 1A). Of note, 386 genes were uniquely expressed in the BLV+ samples and 958 in the BLV− samples. The cluster analysis (Figure 1B) provided a graphical representation of the DEGs across all six samples and illustrated the stark differences between the BLV+ and BLV− groups, mainly when comparing the BLV− cows with the moderate- and high-PVL cows. The same graph (Figure 1B) displayed clusters of genes in the low-PVL cow that differed from the moderate- and high-PVL groups which showed nearly identical expressions. 

### 3.2. Functional Analysis

**Figure 2:**
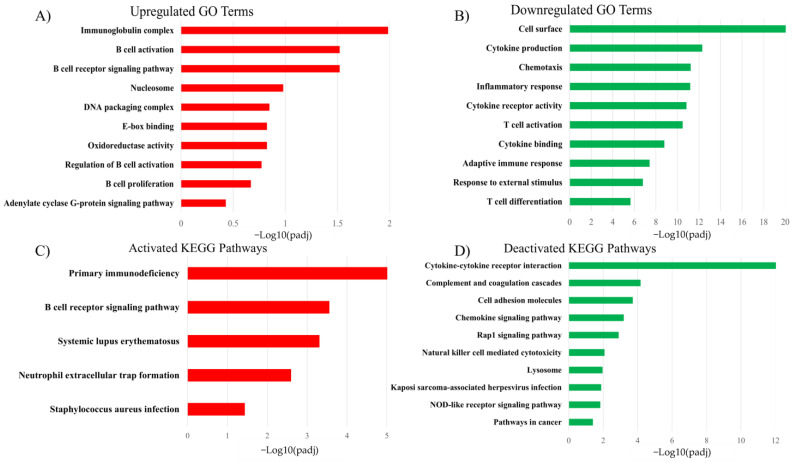
Bar graphs display the most significant functional analysis terms identified from the BLV+ vs. BLV− DEGs using the Gene Ontology and KEGG enrichment analyses. (**A**) Upregulated GO terms in BLV+ PBMCs. (**B**) Downregulated GO terms in BLV+ PBMCs. (**C**) Activated KEGG pathways in BLV+ PBMCs. (**D**) Suppressed KEGG pathways in BLV+ PBMCs.

The Gene Ontology enrichment analysis identified three significantly upregulated GO terms in BLV+ PBMCs (Figure 2A) including the B cell receptor signaling pathway, B cell activation, and immunoglobulin complex. There were 785 significantly downregulated GO terms (Figure 2B) identified in BLV+ PBMCs including cytokine production, cell chemotaxis, T cell activation, and inflammatory response. The KEGG enrichment analysis identified five upregulated KEGG pathways (Figure 2C) including primary immunodeficiency and the B cell receptor signaling pathway in PBMCs from the BLV+ animals. There were 26 downregulated KEGG pathways (Figure 2D) in BLV+ PBMCs including cytokine–cytokine receptor interaction, complement and coagulation cascades, and cell adhesion molecules.

## 4. Discussion

### 4.1. Study and Limitations

This study utilized a novel approach to investigate systemic changes in immune function during BLV infection by analyzing the differential gene expression of the PBMC bulk transcriptomes from BLV-positive and -negative cows. This strategy resulted in the identification of hundreds of DEGs and provided insight into the specific biological pathways impacted by these differences in gene expression. This method allowed for a more thorough examination of immune expression in BLV-infected animals than in previous work; however, there are several limitations in this study. Quantitative PCR was not used in conjunction with RNA sequencing, which could have added data to reinforce the results. The sample size was powered to provide significantly differentially expressed genes but was low enough that the results could be skewed by biological outliers not representative of the norm. The seronegative samples were not confirmed negative with PCR, leaving the possibility that the controls could be PCR-positive and not yet seroconverted [16]. There was no longitudinal aspect to address immune dysfunction at different stages of disease progression in an individual. Other systemic variables such as complete blood count were not analyzed. These data could have potentially provided stronger support for the conclusions or identified abnormalities in the animals. 

### 4.2. Suppression of Immune Response

The majority of the downregulated DEGs in the BLV-infected cows point to an overall suppression of essential immune processes such as cytokine and chemokine activity (e.g., *CX3CR1*, *TGFB2*, and *IL21R*), cell migration (e.g., *CMKLR1*, *CCR3*, and *CCL5*), NOD-like receptor signaling (e.g., *CARD9*, *IKBKE*, and *NLRC4*), and adhesion molecule expression (e.g., *CADM1*, *ICAM3*, and *JAML*) (Table 1). These genes and pathways are critical in mounting an adequate inflammatory response to pathogens and other stressors through the activation and recruitment of immune cells [17,18,19,20]. Downregulation in these groups provides transcriptional evidence that supports previous studies that identified irregular immune profiles in BLV-infected animals [4,21,22]. Some of the recognized pathways and DEGs concerning immune regulation are very similar to the results of previous gene expression work utilizing microarray analysis [23]. However, RNA sequencing’s higher sensitivity and broader range of detection allowed for a more extensive identification of DEGs in the current study. Further, these transcriptomic results present a comprehensive biological basis for previous studies, demonstrating a higher incidence of infectious disease in BLV-infected animals [24,25].

### 4.3. Upregulation of Chemokine Interferon Gamma-Induced Protein 10

Almost no immune cell signaling molecule-coding genes were upregulated in the BLV-positive samples save for one, the chemokine interferon gamma-induced protein 10 (*CXCL10;* Padj 2.55 × 10^−4^; Log2 fold change 1.7). The upregulation of this gene has been associated with the pathogenesis of many diseases and autoimmune disorders in humans [26]. Likewise, *CXCL10* has been proposed as a biomarker to monitor the infection progression of the retroviruses human T-lymphotropic virus 1 (HTLV-1) and human immunodeficiency virus [27,28]. *CXCL10* is also active in B cell lymphoma and leukemia and high circulating concentrations predict unfavorable clinical outcomes [29,30]. Because of its significance as a biomarker for several diseases similar to BLV infection, *CXCL10* should be a gene of interest in further studies to better understand its function in BLV disease progression and explore its viability as a biomarker. 

### 4.4. Altered B and T Cell Gene Expression

Many DEGs and associated differentially expressed pathways observed in our data affect specific PBMC subtypes. The downregulation of the pathways concerning T cell activation and differentiation that include, for example, the genes *ZAP70*, *TAGAP*, and *TIGIT* [31,32,33] are indicative of decreased T cell activity in BLV+ cattle. This, combined with the upregulation of *HAVCR2* (Padj 1.09 × 10^−10^; Log2 fold change 1.5) (TIM-3), a T cell inhibitory molecule, further points to an irregular immune profile indicating the inhibition of T cell activity, a crucial aspect of adaptive immunity. This downregulation reinforces previous work that has identified abnormal T cell phenotypes and protein expression in cows with BLV [34,35,36,37].

The upregulation of the genes critical to B cell functions like B cell activation (e.g., *BTK*, *FCRL1*, and *FCRL3*) and B cell receptor signaling (e.g., *BLNK*, *SYK*, *CD79A*, and *CD79B*) pathways [38,39,40] shows heightened B cell activity and may be evidence that BLV induces unregulated polyclonal B cell proliferation associated with persistent lymphocytosis present in 30% of the infected animals. Interestingly, the gene *FCGR2B* (CD32B), an inhibitory receptor found on several immune cells, was upregulated (Table 1) in the samples from the BLV+ cows. *FCGR2B* regulates B cell activation and suppresses dendritic cell and B cell antigen presentation to T cells [41]. The upregulation of this gene further supports the evidence of decreased T cell activation due to the possible suppression of antigen presentation. 

### 4.5. BLV Pathogenesis

The suppression of the genes underlying T cell activation, cytokine–cytokine receptor interaction, and the activation of the genes responsible for B cell-mediated T cell suppression indicates altered immune cell crosstalk. This abnormal cell communication could disrupt typical signal cascades, ultimately leading to the systemic signs of immunodeficiency and reduced inflammatory response genes observed in these data. The results from the cluster analysis suggest that disease state and PBMC gene expression may vary depending on PVL levels. However, the sample size was not powered to confirm this assumption and requires further studies to draw definitive conclusions. These results support previous studies that have identified a correlation between clinical manifestation or immunological markers and PVL [12,42,43]. Further investigation into this relationship could potentially reveal markers for the early detection of infection and lead to a more comprehensive understanding of how the disease progresses. 

## 5. Conclusions

Our differential gene expression analysis of the bulk transcriptome of PBMCs from BLV-positive and -negative cows has identified genes with potential roles in the development and progression of immune dysfunction in BLV-infected animals. Many of these genes have not previously been associated with BLV or investigated in the context of BLV disease progression and offer new targets for further study of immune dysfunction in BLV infection. Our findings support the association between BLV infection and changes in B cell and T cell gene expression profiles and indicate potential transcriptional changes in innate immune responses suggestive of immunosuppression.

## Figures and Tables

**Table 1:** Most significant BLV+ vs. BLV− DEGs selected based on primary function.

Functional Group	Gene Symbol	Log2 Fold Chamge	Padj	Gene Symbol	Log2 Fold Change	Padj
B cell Activity	*BLNK*	1.74	8.56 × 10^−14^	*CD79A*	1.45	1.70 × 10^−7^
	*SYK*	1.27	2.25 × 10^−13^	*POU2F2*	1.09	3.06 × 10^−7^
	*BANK1*	1.80	5.93 × 10^−12^	*FCRL3*	1.63	1.29 × 10^−6^
	*CD79B*	1.89	1.45 × 10^−11^	*CD22*	1.15	1.46 × 10^−6^
	*TNFRSF13*	1.95	2.26 × 10^−11^	*RFTN1*	1.07	4.49 × 10^−6^
	*FCRL1*	1.32	7.58 × 10^−8^	*CD320*	1.32	5.01 × 10^−6^
	*BTK*	1.05	1.12 × 10^−7^	*VPREB3*	1.98	1.06 × 10^−5^
	*FCGR2B*	1.28	1.62 × 10^−7^	*GPR183*	1.08	1.11 × 10^−5^
T cell Activity	*ITGAL*	−1.63	2.58 × 10^−13^	*BCL6*	−1.98	2.74 × 10^−7^
	*IL6ST*	−1.56	3.70 × 10^−9^	*ZAP70*	−1.41	3.13 × 10^−7^
	*IDO1*	−1.97	4.35 × 10^−9^	*IL7R*	−1.57	9.66 × 10^−7^
	*SEMA4A*	−1.67	7.02 × 10^−9^	*FYN*	−1.52	1.33 × 10^−6^
	*CCDC88B*	−1.68	1.45 × 10^−8^	*PLCG1*	−1.23	2.38 × 10^−6^
	*SPN*	−1.58	3.87 × 10^−8^	*TAGAP*	−1.20	1.19 × 10^−5^
	*CD8A*	−1.41	2.08 × 10^−7^	*TIGIT*	−1.39	1.99 × 10^−5^
Cytokine and Chemokine Activity PAMP Signaling and Cell Surface Molecules	*CX3CR1*	−2.07	3.36 × 10^−18^	*PADI2*	−2.73	7.96 × 10^−9^
	*ADGRG1*	−2.07	2.96 × 10^−17^	*NOTCH1*	−1.07	3.04 × 10^−8^
	*KLRK1*	−2.05	2.14 × 10^−16^	*ITGA5*	−1.60	5.34 × 10^−8^
	*TGFB2*	−5.74	4.09 × 10^−16^	*ARRB2*	−1.34	1.07 × 10^−7^
	*IL21R*	−1.84	1.40 × 10^−12^	*CCR4*	−1.96	1.66 × 10^−7^
	*CMKLR1*	−2.64	9.07 × 10^−12^	*FCGR3A*	−1.71	2.30 × 10^−7^
	*CARD9*	−2.35	1.20 × 10^−11^	*ADAM15*	−1.21	2.95 × 10^−7^
	*ENG*	−2.11	7.38 × 10^−11^	*TNFRSF1B*	−1.44	3.16 × 10^−7^
	*LY6G6C*	−3.00	2.06 × 10^−10^	*TLR3*	−1.46	3.87 × 10^−7^
	*LCP2*	−1.34	2.12 × 10^−10^	*CD226*	−2.09	1.45 × 10^−6^
	*C3*	−1.89	1.65 × 10^−9^	*ICAM3*	−1.39	2.70 × 10^−6^
	*CCR3*	−2.33	2.11 × 10^−9^	*JAML*	−1.37	3.55 × 10^−6^
	*PLCB2*	−1.19	2.41 × 10^−9^	*IKBKE*	−1.37	4.97 × 10^−6^
	*CCL5*	−1.45	3.29 × 10^−9^	*NLRC4*	−1.72	4.54 × 10^−5^

## Data Availability

The data supporting the conclusions of this article will be made available by the authors upon request.
